# HER2-positive apocrine carcinoma of the breast: a population-based analysis of treatment and outcome

**DOI:** 10.1007/s10549-022-06578-4

**Published:** 2022-03-30

**Authors:** Faruk Skenderi, Mohamad Alhoda Mohamad Alahmad, Emin Tahirovic, Yaman M. Alahmad, Zoran Gatalica, Semir Vranic

**Affiliations:** 1 Faculty of Health Sciences, University of Sarajevo, Sarajevo, Bosnia and Herzegovina; 2 Kansas University Medical Center, Kansas City, KS USA; 3 Faculty of Engineering and Natural Sciences, International University of Sarajevo, Sarajevo, Bosnia and Herzegovina; 4 College of Medicine, QU Health, Qatar University, PO Box 2713, Doha, Qatar; 5 Medical Education, Hamad Medical Corporation, Doha, Qatar; 6 Department of Pathology, University of Oklahoma Health Sciences Center, Oklahoma City, OK USA

**Keywords:** Breast cancer, Special types, Apocrine carcinoma, HER2, Outcome

## Abstract

**Purpose:**

Apocrine carcinoma of the breast (APO) expresses HER2 in 30–50% of cases. This study explored the clinicopathological features and outcome of HER2+/APO and matched HER2+/NST cohort.

**Methods:**

We used the SEER database to explore the cohorts. Univariate and multivariate analyses were used to assess the survival. Based on ER and PR [steroid receptors/SR/] and HER2 status, we divided the cohorts to match the intrinsic molecular subtypes for comparisons.

**Results:**

We retrieved 259 cases of HER2+/APO. Most HER2+/APO were SR negative (65%). HER2+/APO were more prevalent in the 80+ age group (24.7% vs. 15.7%, *p* < 0.001). HER2+/SR−/APO had a significantly lower histological grade than the HER2+/SR−/NST (*p* < 0.001). Breast cancer-related deaths were more prevalent in HER2+/NST (7.8% vs. 3.9%, *p* = 0.019). This was particularly evident between SR− subgroups (10.4% in HER2+/SR−/NST vs. 4.2% in HER2+/SR−/APO, *p* = 0.008) and was reaffirmed in breast cancer-specific survival in univariate analysis (*p* = 0.03). Other than race and SR status, HER2+/APO subgroups did not differ in clinicopathological parameters.

**Conclusions:**

Our study confirms the rarity of the APO and reveals that SR status in APO does not affect these patients' prognosis. HER2+/APO tumors tend to have a less aggressive phenotype and a more favorable outcome despite a markedly lower ER/PR positivity.

## Introduction

Breast carcinoma with apocrine differentiation or apocrine carcinoma (APO) is a rare subtype of invasive breast cancer (~ 1%) that has a characteristic apocrine morphology along with the androgen receptor (AR) expression and the lack of estrogen receptor (ER) activity [3, 14, 21]. Based on the proposed classification, all APO are either triple-negative (50–70%) or HER2-positive (30–50%) [3, 14, 18, 20–22].

The available clinical data on APO are contradictory due to the rarity of the disease and the use of different diagnostic criteria for APO [14, 19, 21]. Several recent studies have reported a worse clinical outcome in patients with APO than invasive breast carcinoma of no special type (IBC NST) [4, 24]. However, in an analysis of the Surveillance, Epidemiology, and End Results (SEER) population-based data, the clinical outcome for the APO patients was significantly better than NST patients following the adjustment for demographic and clinicopathological characteristics [24]. Some studies also revealed more favorable overall survival (OS) and breast cancer-specific survival (BCSS) for patients with AR-positive triple-negative APO compared with other triple-negative breast carcinomas (TNBC) [10–12, 25]. In contrast, other studies found no significant differences [5, 13]. Wu et al. recently reported better clinical outcomes of triple-negative APO patients than TNBC NST [23]. The authors also found chemotherapy associated with a more favorable outcome among the triple-negative APO patients [23].

Despite the common HER2 expression, most of the available clinical studies have been focused on the triple-negative APO, while the clinical and survival data on HER2+/APO are sparse [7, 8]. The current study explored the association between APO subtypes and survival after adjusting for all other prominent clinical and demographic predictors of survival among SEER patients.

## Materials and methods

### Patients' selection and cohorts

We explored the National Cancer Institute’s Surveillance, Epidemiology, and End Results (SEER) database to select our study cohort. The SEER database includes data on patients' demographics, tumor characteristics [Histotype, tumor grade, TNM stage (AJCC), tumor size, lymph node status, and distant metastases], the first course of treatment, treatment options (surgery, chemotherapy, radiotherapy), and follow-up for vital status. It encompasses data from eighteen population-based cancer registries covering approximately 1/3 of the US population.

For our study cohort, we selected patients diagnosed with histologically confirmed invasive HER2-positive APO (HER2+/APO) between 2010 and 2016. In addition, we selected patients diagnosed with IBC NST with HER2 positivity (HER2+/NST) within the same period. The two groups (HER2+/APO and HER2+/NST) were further divided based on the SEER variable that codes ER and Progesterone Receptor (PR) [Steroid receptors/SR/] and HER2 status to form groups based on the intrinsic molecular subtypes, i.e., Apocrine Luminal B (HER2+/SR+/APO) and Apocrine HER2-Enriched (HER2+/SR−/APO) for HER2+/APO and HER2+/SR+/NST and HER2+/SR−/NST for HER2+/NST part of the cohort. SR status did not include androgen receptor. The study excluded in situ carcinomas (both apocrine and NST) and other special types of invasive breast carcinoma with HER2 positivity. We used the SEER*stat version 8.3.2 to generate a case-listing file.

### Statistical analysis

Patients’ demographical and clinicopathological characteristics, including age, hormone receptor status, stage, tumor grade, therapies (surgery, chemotherapy, radiotherapy), regional and distant metastases, as well as metastatic sites, were summarized by absolute and relative frequencies. Age was categorized as 18–49, 50–79, and ≥ 80 years. The difference in the distribution of clinicopathological characteristics was tested using Pearson Chi-square and/or Fisher's exact test, whichever was appropriate according to the expected cell counts. For continuous normally distributed variables, we give mean and standard deviation as measures of central tendency and variability; for those whose distribution is not normal, we give a median and interquartile range instead. We tested the equality of means/medians for normally/non-normally distributed variables using the *t* test/Mann–Whitney *U* test.

BCSS and OS were defined as a time in years between the cancer diagnosis and death from breast cancer, death due to any cause, respectively. Patients alive at the end of the follow-up were censored for both types of survival. We ran univariate Cox proportional hazards analysis to identify candidate prognostic factors for the multivariate analysis. For each of these, we produced Kaplan–Meier plots and performed log-rank tests after checking the appropriateness of proportional hazard assumption graphically using martingale and weighted Schoenfeld residuals. After identifying and deciding which prognostic factors will enter the multivariate analyses, we fitted a multivariate Cox regression model to estimate the effect of subtypes on survival while adjusting for other prognostic factors. In the multivariate analysis of BCSS and OS, aside from the HER2+/APO status, we included age (categorical), stage, tumor grade, surgery, and systemic therapies. To provide an additional check of the robustness of our findings w.r.t. modeling assumptions assumed for multivariate Cox regression, we applied propensity score caliper matching in the ratio of 3:1 in favor of the “control” group (HER2+/NST). The propensity score was estimated via a multivariate logistic regression model that included age (in years, continuous), tumor grade, stage, size (in cm), lymph node status, the presence of distant metastasis, chemotherapy and radiation therapy. We applied the same model for propensity score and the same matching strategy for APO vs. NST comparisons (overall, SR+ and SR−) and the analysis for the effect of SR status among the APO subtype. All analyses were performed based on available cases using R Statistical Software (Foundation for Statistical Computing, Vienna, Austria) version 3.5.1. All tests were 2-sided, with *p* < 0.05 considered statistically significant.

## Results

### HER2+/APO vs. HER2+/NST cohorts

The clinicopathological characteristics of the cohorts are summarized in Table 1.

**Table 1:** Clinicopathological characteristics of HER2+ breast cancer patients from the SEER database by histological subtype

Variable	Entire sample *N* (%)	APO *n* (%)	NST *n* (%)	*p*-value—Pearson Chi Square (Fisher exact)
*Age*				**< 0.001 (0.002)**
18–49	6722 (12.7)	23 (8.9)	6699 (12.7)	
50–79	38,050 (71.6)	172 (66.4)	37,878 (71.7)	
≥ 80	8347 (15.7)	64 (24.7)	8283 (15.7)	
*Age mean (SD)*				** < 0.001**
	65.35 (13.79)	69.1 (14.27)	65.3 (13.78)	
*Race*				0.992 (0.994)
Black	6795 (12.8)	33 (12.7)	6762 (12.8)	
White	39,720 (74.8)	194 (74.9)	39,526 (74.8)	
Other	6214 (11.7)	31 (12)	6183 (11.7)	
NA	390 (0.7)	1 (0.4)	389 (0.7)	
*ER/PR status (SR)*				** < 0.001 (< 0.001)**
Negative	16,353 (30.8)	167 (64.5)	16,186 (30.6)	
Positive	36,647 (69)	91 (35.1)	36,556 (69.2)	
NA	119 (0.2)	1 (0.4)	118 (0.2)	
*Grade*				0.492 (0.512)
I	2262 (4.3)	10 (3.9)	2252 (4.3)	
II	17,870 (33.6)	96 (37.1)	17,774 (33.6)	
III	30,309 (57.1)	140 (54.1)	30,169 (57.1)	
NA	2678 (5)	13 (5)	2665 (5)	
*Stage*				0.489 (0.450)
I	19,753 (37.2)	105 (40.5)	19,648 (37.2)	
II	19,905 (37.5)	86 (33.2)	19,819 (37.5)	
III	7925 (14.9)	42 (16.2)	7883 (14.9)	
IV	4181 (7.9)	22 (8.5)	4159 (7.9)	
NA	1355 (2.6)	4 (1.5)	1351 (2.6)	
*Tumor size (cm)*				0.731 (0.758)
< 2	21,939 (41.3)	108 (41.7)	21,831 (41.3)	
2–5	22,211 (41.8)	116 (44.8)	22,095 (41.8)	
> 5	5439 (10.2)	24 (9.3)	5415 (10.2)	
NA	3530 (6.6)	11 (4.2)	3519 (6.7)	
*RLN status*				0.292 (0.309)
Negative	30,564 (57.5)	158 (61)	30,406 (57.5)	
Positive	21,694 (40.8)	98 (37.8)	21,596 (40.9)	
NA	861 (1.6)	3 (1.2)	858 (1.6)	
*Distant metastasis*				0.379 (0.334)
No	48,890 (92)	235 (90.7)	48,655 (92)	
Yes	3662 (6.9)	19 (7.3)	3643 (6.9)	
Unknown	567 (1.1)	5 (1.9)	562 (1.1)	
*Cause of death*				**0.019 (0.018)**
Breast cancer	4109 (7.7)	10 (3.9)	4099 (7.8)	
Other	49,010 (92.3)	249 (96.1)	48,761 (92.2)	
*Surgery performed*				0.538 (0.465)
No	5490 (10.3)	25 (9.7)	5465 (10.3)	
Yes	46,663 (87.8)	227 (87.6)	46,436 (87.8)	
Unknown	966 (1.8)	7 (2.7)	959 (1.8)	
*Chemotherapy*				0.413 (0.403)
No/unknown	14,591 (27.5)	77 (29.7)	14,514 (27.5)	
Yes	38,528 (72.5)	182 (70.3)	38,346 (72.5)	
*Radiation*				**0.0508 (0.0518)**
No/unknown	29,829 (56.2)	161 (62.2)	29,668 (56.1)	
Yes	23,290 (43.8)	98 (37.8)	23,192 (43.9)	
*Vital status*				0.097 (0.101)
Alive	46,845 (88.2)	237 (91.5)	46,608 (88.2)	
Dead	6274 (11.8)	22 (8.5)	6252 (11.8)	
*Follow up months median (IQR)*				**0.038**
	31 (39)	27 (35.5)	31 (39)	

*NST* no special type, *APO* apocrine, *NA* not available, *SR* steroid receptors (ER and PR), *RLN* regional lymph node, *IQR* interquartile range

Only significant *p*-values are bolded

Among 446,806 breast malignancies diagnosed between 2010 and 2016, there were 259 with HER2+ APO subtype (HER2+/APO) (~ 0.06%). We also identified 52,860 HER2+ IBC NST (HER2+/NST) in the same period. The median follow-up for the pooled cohort was 31 months (14 = 1st quartile, 53 = 3rd quartile); the median follow up for HER2+/APO was 27 months (12.5 = 1st quartile, 48 = 3rd quartile) and 31 months (14 = 1st quartile, 53 = 3rd quartile) for HER2+/NST cohort (*p* = 0.038, Table 1).

The average age of patients was 69.1 years in the HER2+/APO group and 65.3 years in the HER2+/NST group (*p* < 0.001). HER2+/APO were more prevalent in 80+ age group (24.7%, *n* = 64) compared with HER2+/NST cohort (15.7%, *n* = 8283) (*p* < 0.001). HER2+/APO had a significantly lower SR (ER and PR) positivity than the HER2+/NST group (*p* < 0.001) (Table 1). AR status was not routinely reported in the SEER database.

The two groups did not differ significantly regarding the race, tumor stage, regional lymph node status or the presence of distant metastases (in general and in specific sites). The treatment options (surgery and chemotherapy) were similar between HER2+/APO and HER2+/NST (*p* > 0.05), while radiotherapy was more commonly used in the HER2+/APO group compared with HER2+/NST (*p* = 0.05) (Table 1).

**Fig. 1:**
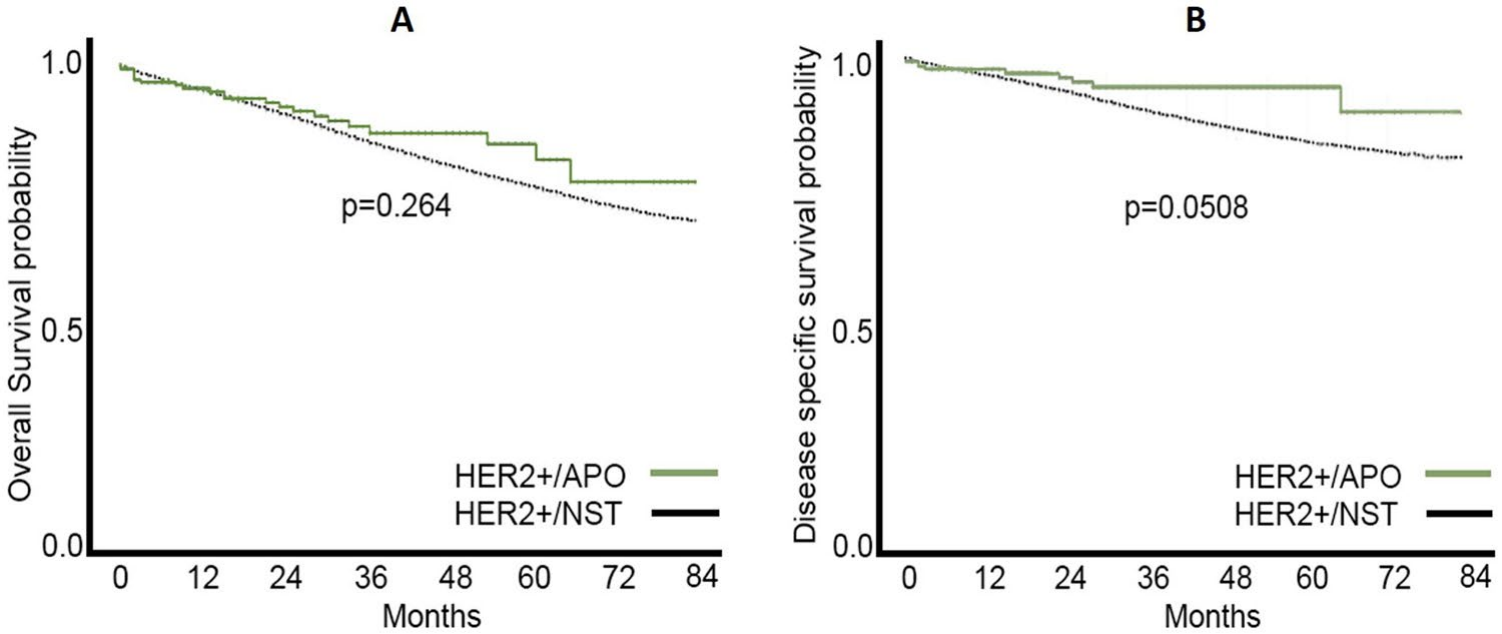
**A**, **B** HER2+/APO had seemingly better OS than HER2+/NST patients (**A**); however, the difference was not statistically significant (*p* = 0.264). Nevertheless, this effect was better seen in BCSS and almost reached a statistical significance (*p* = 0.0508) (**B**)

Although at 18 months and onwards the OS estimate in the HER2+/APO group was consistently higher, with unadjusted HR 1.26 (0.83, 1.92) in the univariate Cox proportional hazards model. This difference was not statistically significant (*p* = 0.264) (Fig. 1A). Nevertheless, the positive effect of apocrine morphology was more evident in BCSS analysis with unadjusted HR 1.83 (0.99, 3.42), hence 83% higher hazard for BCSS death in the HER2+/NST group (Fig. 1B).

Breast cancer as a cause of death was also more prevalent in HER2+/NST than in the HER2+/APO group (7.8% vs. 3.9%, *p* = 0.019). This difference was 1.5 times higher between ER-negative subgroups (4.2% in HER2+/SR−/APO vs. 10.4% in HER2+/SR−/NST, *p* = 0.008). It was reflected in the results of the univariate analysis of BCSS in those groups with unadjusted HR 2.22 (1.06, 4.67) and adjusted (multivariate analysis) HR 2.07 (0.93, 4.62).

**Fig. 2:**
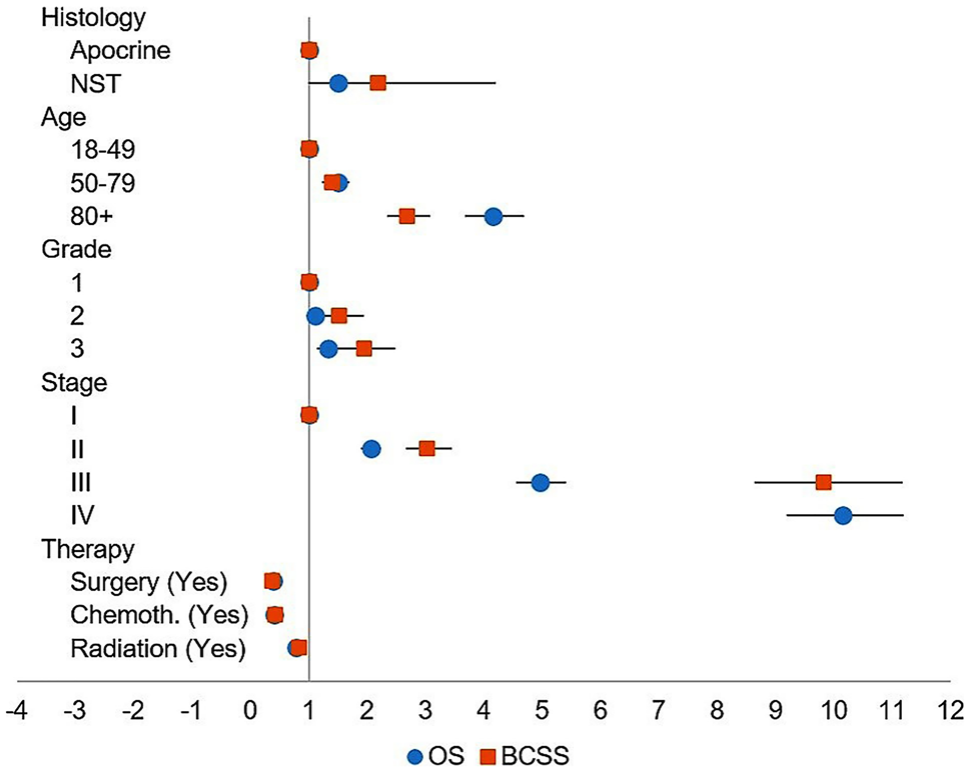
Overall and breast cancer-specific survival Hazard ratios. Multivariate Cox Proportion Hazards model analysis of HER2+/APO vs. HER2+/NST group. Apocrine morphology in HER2+breast cancer confers a lower hazard ratio than NST morphology (*p* = 0.018)

In a multivariate Cox proportional hazards model analysis, HER2+/APO patients had better OS (HR = 1.512, CI = 0.9–2.32, *p* = 0.059) and significantly better BCSS (HR = 2.19, CI 1.13–4.21, *p* = 0.018) compared with HER2+/NST (Table 2, Fig. 2).

**Table 2:** Multivariate cox proportional hazards model analysis of HER2+/APO vs. HER2+/NST group

Variable	OS				BCSS			
	HR	95% CI		*p*-value	HR	95% CI		*p*-value
*Histology*								
Apocrine	1.000							
NST	1.512	0.984	2.321	**0.059**	2.1913	1.1388	4.2168	**0.018**
*Age*								
18–49	1.000							
50–79	1.502	1.339	1.685	**0.000**	1.3956	1.2313	1.5817	**0.001**
≥ 80	4.156	3.685	4.687	**0.000**	2.6913	2.3473	3.0858	**0.001**
*Grade*								
1	1.000							
2	1.117	0.958	1.301	1.000	1.5249	1.1901	1.954	**0.001**
3	1.336	1.150	1.553	**0.000**	1.9483	1.5255	2.4882	**0.001**
*Stage*								
I	1.000							
II	2.062	1.900	2.238	**0.000**	3.0315	2.6642	3.4494	**0.001**
III	4.964	4.551	5.415	**0.000**	9.8249	8.6379	11.1751	**0.001**
IV	10.147	9.193	11.201	**0.000**	23.3762	20.3544	26.8465	**0.001**
*Therapy*								
Surgery (yes)	0.385	0.357	0.415	**0.000**	0.3689	0.3375	0.4032	**0.001**
Chemotherapy (yes)	0.414	0.390	0.439	**0.000**	0.4288	0.3976	0.4625	**0.001**
Radiation (yes)	0.788	0.741	0.838	**0.000**	0.8452	0.7839	0.9113	**0.001**

Only significant *p*-values are bolded

*NST* no special type, *OS* overall survival, *BCSS* breast cancer-specific survival, *HR* hazard ratio, *CI* confidence interval

### HER2+/SR−/APO vs. HER2+/SR+/APO cohorts

The clinicopathological characteristics of the HER2+/APO cohort are summarized in Table 3.

**Table 3:** Clinicopathological characteristics of the HER2+ apocrine cohort (SR+ vs. SR−)

Variable	Entire sample (*N* and %)	HER2+/ER/PR−/APO (*N* and %)	HER2+/ER/PR+/APO (*N* and %)	*p*-value—Pearson Chi-Square (Fisher exact)
*Age*				0.893 (0.91)
18–49	23 (8.9)	14 (8.4)	8 (8.8)	
50–79	172 (66.4)	110 (65.9)	62 (68.1)	
≥ 80	64 (24.7)	43 (25.7)	21 (23.1)	
*Age (mean), years (SD)*				0.424
	69.12 (14.27)	69.7 (14.33)	68.3 (14.06)	
*Race*				**0.022 (0.018)**
Black	33 (12.7)	17 (10.2)	16 (17.6)	
White	194 (74.9)	123 (73.7)	70 (76.9)	
Other	31 (12)	26 (15.6)	5 (5.5)	
NA	1 (0.4)	1 (0.6)	0 (0)	
*Estrogen receptor*				** < 0.001 (< 0.001)**
Negative	173 (66.8)	167 (100)	6 (6.6)	
Positive	85 (32.8)	0 (0)	85 (93.4)	
NA	1 (0.4)	0 (0)	0 (0)	
*Progesterone receptor*				** < 0.001 (< 0.001)**
Negative	201 (77.6)	167 (100)	34 (37.4)	
Positive	56 (21.6)	0 (0)	56 (61.5)	
NA	2 (0.8)	0 (0)	1 (1.1)	
*Grade*				0.098 (0.099)
1	10 (3.9)	9 (5.4)	1 (1.1)	
2	96 (37.1)	56 (33.5)	40 (44)	
3	140 (54.1)	93 (55.7)	47 (51.6)	
NA	13 (5)	9 (5.4)	3 (3.3)	
*Stage*				0.889 (0.880)
I	105 (40.5)	69 (41.3)	35 (38.5)	
II	86 (33.2)	54 (32.3)	32 (35.2)	
III	42 (16.2)	28 (16.8)	14 (15.4)	
IV	22 (8.5)	13 (7.8)	9 (9.9)	
NA	4 (1.5)	3 (1.8)	1 (1.1)	
*Tumor size (cm)*				0.917 (0.912)
< 2	108 (41.7)	69 (41.3)	38 (41.8)	
2–5	116 (44.8)	77 (46.1)	39 (42.9)	
> 5	24 (9.3)	15 (9)	9 (9.9)	
NA	11 (4.2)	6 (3.6)	5 (5.5)	
*RLN status*				0.333 (0.349)
Negative	158 (61)	98 (58.7)	59 (64.8)	
Positive	98 (37.8)	67 (40.1)	31 (34.1)	
NA	3 (1.2)	2 (1.2)	1 (1.1)	
*Distant metastasis*				0.536 (0.619)
No	235 (90.7)	152 (91)	82 (90.1)	
Yes	19 (7.3)	11 (6.6)	8 (8.8)	
NA	5 (1.9)	4 (2.4)	1 (1.1)	
*Cause of death*				0.722 (1)
Breast	10 (3.9)	7 (4.2)	3 (3.3)	
Other	249 (96.1)	160 (95.8)	88 (96.7)	
*Surgery performed*				0.778 (0.843)
No	32 (12.4)	20 (12)	12 (13.2)	
Yes	227 (87.6)	147 (88)	79 (86.8)	
*Chemotherapy*				0.097 (0.1161)
No/unknown	77 (29.7)	55 (32.9)	21 (23.1)	
Yes	182 (70.3)	112 (67.1)	70 (76.9)	
*Radiation*				0.5135 (0.5915)
No/unknown	161 (62.2)	106 (63.5)	54 (59.3)	
Yes	98 (37.8)	61 (36.5)	37 (40.7)	
*Vital status*				0.4117 (0.49)
Alive	237 (91.5)	151 (90.4)	85 (93.4)	
Dead	22 (8.5)	16 (9.6)	6 (6.6)	
*Survival (months, median and IQR)*				0.199
	27 (12.5, 48)	25(11.45)	28 (16.5, 50)	

*RLN* regional lymph node status, *IQR* interquartile range, *SD* standard deviation, *ER* estrogen receptor, *PR* progesterone receptor, *APO* apocrine carcinoma

Only significant *p*-values are bolded

Among the APO, 167 cases were HER2+/SR− (HER2-enriched) (65%), while 91 patients (35%) were HER2+/SR+ (Luminal B) APO. HER2+/SR+/APO subtype was more prevalent in the Black race, while HER2+/SR− APO were more frequent in other races (neither black nor white) (*p* = 0.022) (Table 3). Notably, there was no significant difference between the two APO subgroups regarding the tumor grade, stage, lymph node status, distant metastases in general and specific organs, cause of death, and therapeutic modalities (*p* > 0.05, for all variables) (Table 3).

**Fig. 3:**
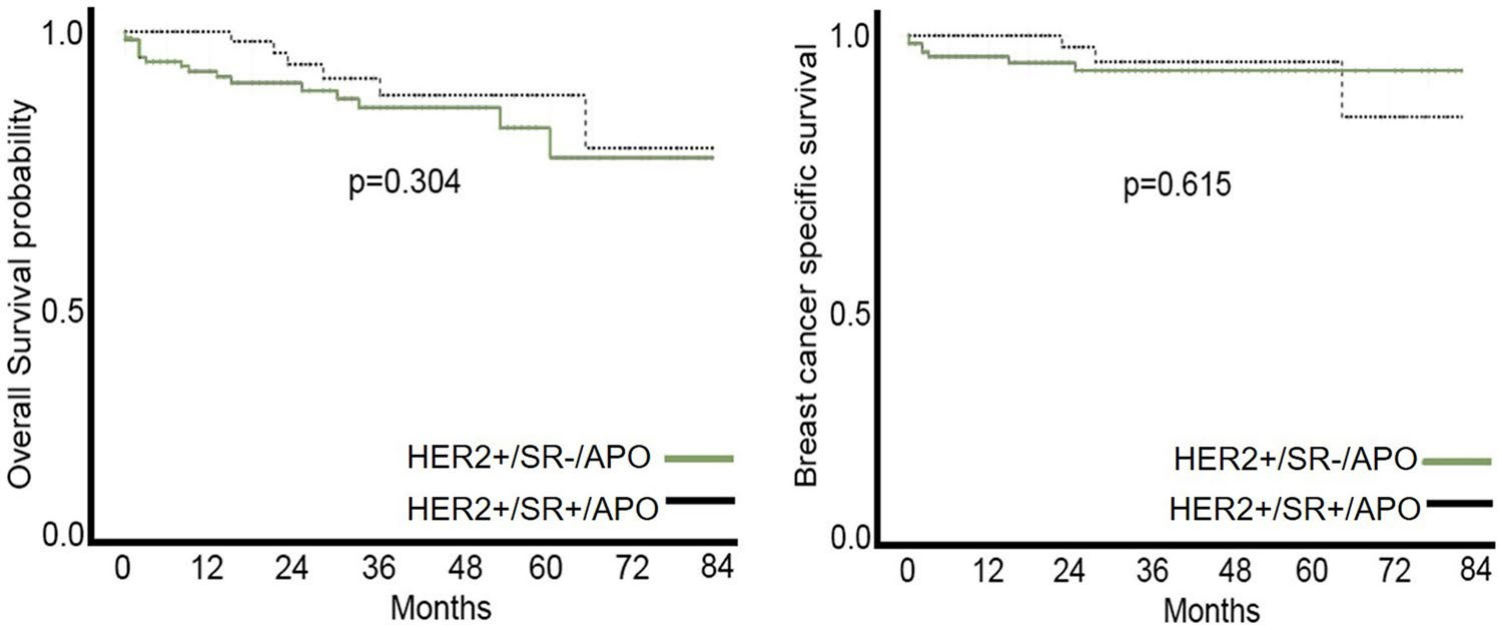
SR (estrogen and progesterone receptors) status had no impact on survival as there was no difference in OS and BCSS between the two APO subgroups (*p* = 0.304 and 0.615, respectively)

The survival curves for two groups defined by SR status (ER and PR) were not significantly different by the log-rank test (Fig. 3), although their shape was detectable by the log-rank test statistic. This was the case for both OS [HR 0.6 (0.54, 1.57)] and BCSS [HR 0.7 (0.18, 2.74)] in favor of SR− positive status. The patients aged ≥ 80 years had worse OS in univariate analysis (*p* < 0.001); however, this age-related difference in survival curves completely dissipated when it came to BCSS (*p* = 0.423).

### HER2+/SR+/APO vs. HER2+/SR+/NST cohorts

There were 91 HER2+/SR+/APO (Luminal B) cases and 36,556 HER2+/SR+ /NST (Luminal B) carcinomas. The mean age in HER2+/SR+/APO was 68.3 years, which was significantly higher than in the HER2+/SR+/NST group (65.2 years, *p* = 0.035).

Except for PR, which was less frequent in the HER2+/SR+/APO cohort, the two groups did not differ in clinicopathological parameters, including the treatment modalities. Although in univariate analysis no significant differences were observed, multivariate Cox proportional hazards analysis revealed that the apocrine morphology in SR+/HER2+ carcinomas was associated with better OS (HR = 2.62, CI = 1.17–5.84, *p* = 0.018) and BCSS (HR = 3.14, CI = 1.01–9.76, *p* = 0.047) (Table 4).

**Table 4:** Multivariate Cox proportional Hazard analysis of the HER2/SR+/APO vs. HER2/SR+/NST cohorts

	OS				BCSS			
	HR	95% CI		*p*-value	HR	95% CI		*p*-value
*Histology*								
Apocrine	1.000				1.000			
NST	2.623	1.176	5.848	**0.018**	3.142	1.012	9.763	**0.047**
*Age*								
18–49	1.000				1.000			
50–79	1.551	1.338	1.798	**0.000**	1.411	1.201	1.659	**0.000**
≥ 80	4.409	3.779	5.144	**0.000**	2.570	2.152	3.069	**0.000**
*Grade*								
1	1.000				1.000			
2	1.139	0.962	1.347	0.130	1.664	1.245	2.226	**0.000**
3	1.306	1.106	1.543	**0.001**	2.028	1.520	2.704	**0.000**
*Stage*								
I	1.000				1.000			
II	2.025	1.831	2.239	**0.000**	3.037	2.571	3.587	**0.000**
III	4.633	4.150	5.172	**0.000**	9.792	8.277	11.585	**0.000**
IV	9.633	8.511	10.903	**0.000**	24.486	20.485	29.267	**0.000**
*Therapy*								
Surgery (yes)	0.391	0.355	0.430	**0.000**	0.364	0.325	0.409	**0.000**
Chemotherapy (yes)	0.419	0.388	0.452	**0.000**	0.425	0.386	0.469	**0.000**
Radiation (yes)	0.729	0.674	0.787	**0.000**	0.768	0.695	0.847	**0.000**

Only significant *p*-values are bolded

*OS* overall survival, *BCSS* breast cancer-specific survival, *NST* no special type, *HR* hazard ratio, *CI* confidence interval

### HER2+/SR− APO vs. HER2+/SR− NST cohorts

There were 167 cases of HER2+/SR−/APO and 16,186 HER2+/SR−/NST carcinomas. At diagnosis, the average age was 69.7 in the HER2+/SR−/APO group vs. 65.6 years in the HER2+/SR−/NST group. HER2+/SR−/APO was more prevalent in older populations than the HER2+/SR−/NST (*p* = 0.001).

HER2+/SR−/APO had a significantly lower histological grade than the HER2+/SR−/NST (55.7% vs. 70.5%, *p* < 0.001). The chemotherapy frequency differed between the groups, with more chemotherapy applied in the HER2+/SR−/NST group (75.5%) compared with HER2-enriched APO (67.1%). The two cohorts did not differ regarding the tumor stage, lymph node, and distant metastases' patterns.

**Fig. 4:**
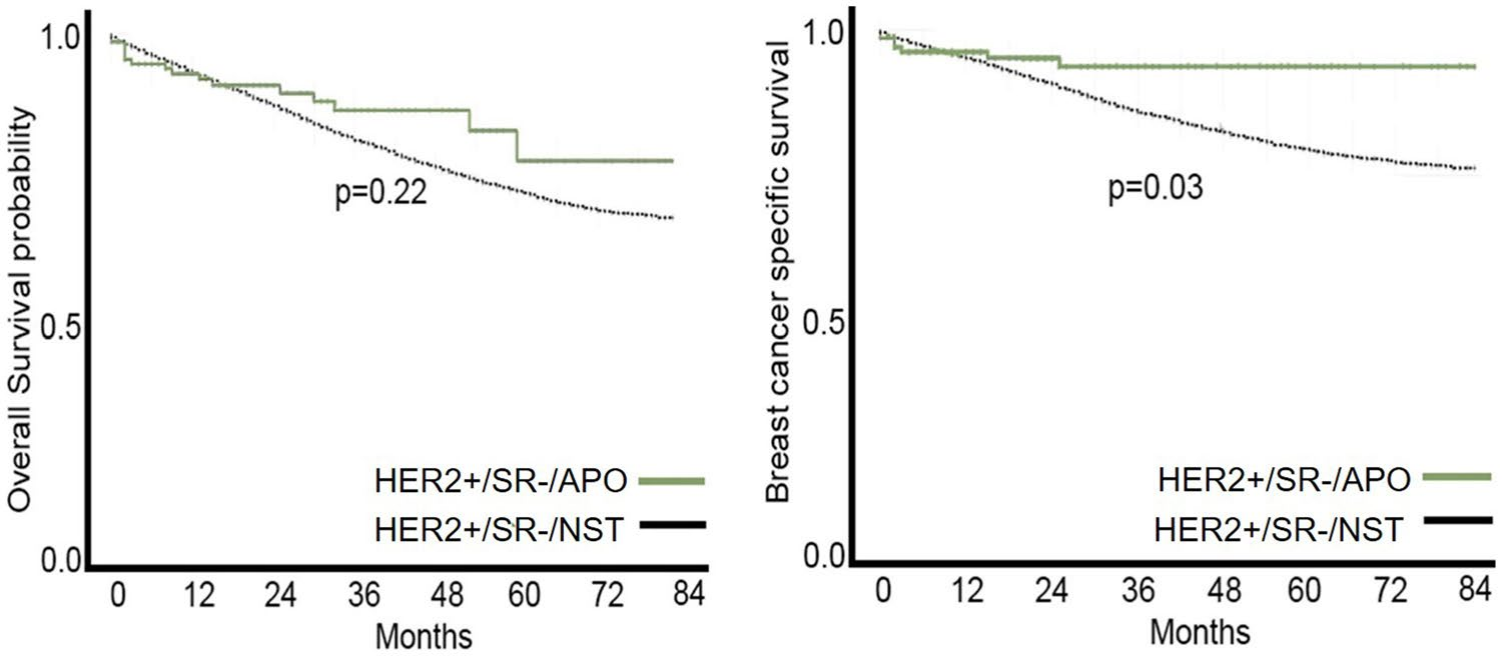
Apocrine morphology in HER2-enriched subgroups was associated with a better outcome in BCSS (*p* = 0.03), while the difference was not seen in the OS analysis (*p* = 0.22)

The cause of death was also significantly different between the groups, as only 4.2% of patients in the HER2+/SR−/APO group died of breast cancer, while 10.4% died of breast cancer in the HER2+/SR−/NST group (*p* = 0.008). Consequently, the apocrine morphology was associated with a better BCSS in univariate analysis (*p* = 0.030) (Fig. 4). However, multivariate analysis did not reveal a significant difference in OS and BCSS between the HER2+/SR−/APO and NST groups.

## Discussion

APO is a rare (frequency ~ 1%) subtype of breast carcinoma whose diagnosis and clinical data, including outcome, remain challenging and controversial [14, 19]. Based on the SEER data (2010–2016), the current study represents the largest HER2-positive APO cohort reported to date.

Our findings indicate that HER2-positive APO do substantially better compared with HER2-positive NST carcinomas. This is mainly reflected in BCSS and was independent of ER and PR status. Our results are in line with a recently published SEER cohort of triple-negative APO, which also revealed that these cancers had a better prognosis than TNBC NST [23]. Several other independent studies confirmed a more favorable outcome for triple-negative APO patients [1, 17]. In contrast, the studies of Dellapasqua et al. [4] and Bonnefoi et al. [2] reported a poor outcome of molecular apocrine tumors/pure APO (as defined by gene expression analysis and/or immunohistochemistry) from the cohorts of the European Institute of Oncology and the EORTC 10,994/BIG 1–00 phase III study, respectively. Saridakis et al. explored the status of APO in the SEER database, reporting a more aggressive clinical course of APO than non-APO but without significant differences in BCSS [16].

Some other findings from our study are consistent with the previous data, including a rarity of APO and a higher prevalence of APO among elderly patients [14, 21, 24]. APO typically lacks ER and PR receptors [6, 14, 21], as confirmed in our study, given that 2/3 of the cases were negative for ER and PR, which was significantly lower than in the HER2+/NST cohort. In contrast, APO consistently overexpress AR [6], but the AR status was not provided in the SEER database. This is understandable given the timeline of the collected data (2010–2016) and the fact that AR testing was only recently incorporated into a recommended diagnostic work-up of the breast's APO [14, 19]. Consequently, some of the tumors in the study, particularly ER/PR-positive, may not be true molecular APO. Nevertheless, our subgroup analysis (HER2-enriched vs. Luminal B APO) revealed no significant differences between the two groups regarding clinicopathological parameters and survival (both OS and BCSS).

We observed some clinically relevant differences between the APO and NST groups. Thus, HER2+/SR−/APO had significantly lower histological grade than the NST carcinomas, which is in line with several previous studies and is probably due to the lower mitotic activity (and lower Ki-67 labeling) of apocrine cells [9, 17, 19, 20]. Lower tumor grade may also contribute to the less aggressive behavior of HER2+/APO, as confirmed in a large cohort of IBS NST in which a high histological grade was a strong predictor of an adverse outcome [15]. In addition to this finding, the more advanced age of HER2+/APO patients probably contributed to substantially lower chemotherapy use in this group of patients. Nevertheless, the chemotherapy use did not affect the outcome, given a significantly better BCSS among HER2-enriched APO than in the HER2-enriched NST cohort.

The current study has several limitations that reflect the nature of the SEER database. Firstly, the follow-up period was too short (~ 31 months), and long-term outcomes could not be assessed. This is particularly relevant for SR+ cases in both cohorts. Secondly, AR was not routinely provided, which could affect the classification of a proportion of HER2+/APO cases, particularly ER/PR-positive. In addition, the details (types and duration) on chemotherapy, endocrine, and anti-HER2 therapies were not provided in the SEER database, so their effects (sensitivity, resistance) and the overall impact on the outcomes could not be analyzed.

We conclude that our cohort represents the largest HER2+/APO study reported to date. It confirmed the rarity of the HER2+/APO and revealed only marginal differences within the HER2+/APO regardless of ER/PR status. Compared with HER2+/NST, HER2+/APO tended to have a less aggressive phenotype and were associated with a more favorable clinical outcome (BCSS) despite a markedly lower/absent ER/PR expression. Further prospective studies should confirm the provided observations.

## Data Availability

The datasets from the study can be obtained from the corresponding author on reasonable request.

## Abbreviations

APO: Apocrine carcinoma (breast carcinoma with apocrine differentiation)
AR: Androgen receptor
BCSS: Breast cancer-specific survival
CI: Confidence interval
ER: Estrogen receptor
HR: Hazard ratio
IQR: Interquartile range
NST: No special type
OS: Overall survival
PR: Progesterone receptor
RLN: Regional lymph nodes
SEER: Surveillance, Epidemiology, and End Results
SR: Steroid receptors (estrogen and progesterone)
TNBC: Triple-negative breast carcinoma
